# The Anti-Inflammatory and Vasodilating Effects of Three Selected Dietary Organic Sulfur Compounds from *Allium* Species

**DOI:** 10.3390/jfb8010005

**Published:** 2017-01-26

**Authors:** Chin-Chen Chu, Wen-Shiann Wu, Ja-Ping Shieh, Heuy-Ling Chu, Chia-Pu Lee, Pin-Der Duh

**Affiliations:** 1 Department of Anesthesiology, Chi-Mei Medical Center, Tainan 71004, Taiwan; chinchen.chu@gmail.com (C.-C.C.); dog.i7007@msa.hinet.net (J.-P.S.); 2 Department of Recreation and Health-Care Management, Chi-Mei Medical Center, Tainan 71004, Taiwan; 3 Division of Cardiology, Chi-Mei Medical Center, Tainan 71004, Taiwan; yyl.yehaline@gmail.com; 4 Center of General Education, Chi-Mei Medical Center, Tainan 71004, Taiwan; 5 Department of Food Science and Technology, Chia Nan University of Pharmacy and Science, Tainan 71710, Taiwan; chuheuy@mail.cnu.edu.tw (H.-L.C.); pulee@mail.cnu.edu.tw (C.-P.L.)

**Keywords:** angiotensin-converting enzyme, anti-inflammatory activity, diallyl disulfide, dimethyl disulfide, propyl disulfide, prostacyclin, vasodilation

## Abstract

The anti-inflammatory and vasodilating effects of three selected dietary organic sulfur compounds (OSC), including diallyl disulfide (DADS), dimethyl disulfide (DMDS), and propyl disulfide (PDS), from *Allium* species were investigated. In the anti-inflammatory activity assay, the three OSC demonstrated significant inhibition of nitric oxide (NO) and prostaglandin E_2_ (PGE_2_) production in LPS-induced RAW 264.7 cells. The expression of inducible nitric oxide synthase (iNOS) and cyclooxygenase (COX-2) in activated RAW 264.7 cells was inhibited by the three OSC, indicating that the three OSC prevented the LPS-induced inflammatory response in RAW 264.7 cells. For the vasodilative assay, the three OSC were ineffective in producing NO in SVEC4-10 cells, but they did enhance prostacyclin (PGI_2_) production. The expression of COX-2 in SVEC4-10 cells was activated by DADS and DMDS. Pretreatment of SVEC4-10 cells with the three OSC decreased ROS generation in H_2_O_2_-induced SVEC4-10 cells. In addition, the three OSC significantly inhibited angiotensin-I converting enzyme (ACE). The up-regulation of PGI_2_ production and COX-2 expression by DADS and DMDS and the reduction of ROS generation by DADS, DMDS, and PDS in SVEC4-10 cells contributed to the vasodilative effect of the three OSC. Collectively, these findings suggest that DADS, DMDS, and PDS are potential anti-inflammatory and vasodilative mediators.

## 1. Introduction

Inflammation is typically characterized by increased permeability of the endothelial tissue and influxes of blood leukocytes into the interstitium, resulting in edema [1]. The activation of leukocytes (e.g., neutrophils and monocytes/macrophage) can produce immune responsive factors, such as nitric oxide (NO), cytokines, and eicosanoid mediators of the immune response [2]. Cytokines produced at appropriate levels against a pathogen infection can be beneficial to the host, but the overexpression of these cytokines can cause serious diseases. Normal inflammatory responses are self-limited by a process that involves the down-regulation of pro-inflammatory mediators and an increase in anti-inflammatory mediators [3]. However, the response of a cell to an increase in oxidants or inflammatory stimuli often involves the activation of numerous intracellular signaling pathways. These cytosolic pathways can regulate a host of transcriptional changes that allow the cell to respond appropriately to oxidative stress and thereby contribute to various pathological conditions [4]. In addition, NO, prostaglandin E_2_ (PGE_2_), and related enzymes have been implicated as important mediators in the inflammation process. The excess production of NO is produced by inducible nitric oxide synthase (iNOS). PGE_2_ is synthesized by cyclooxygenase (COX) (especially COX-2) during inflammation [5]. Therefore, the inhibition of iNOS and COX-2 activity and/or expression is important in all cases of inflammation.

Hypertension is defined as a systolic blood pressure of more than 140 mmHg and a diastolic blood pressure of more than 90 mmHg. It affects up to 30% of the adult population in most countries [6]. High blood pressure increases the risk of heart disease such as arteriosclerosis, stroke, myocardial infarction, and end-stage renal disease [7]. The risk factors for hypertension include obesity, drinking too much alcohol, smoking, and family history. Therefore, lifestyle modifications are one of the most important ways to effectively lower blood pressure [8]. The definition of vasorelaxation is reduction of vascular tension. The vasoactive substances, including NO, prostacyclin (PGI_2_), and endothelium-derived hyperpolarizing factor derived from the endothelium, play an important role in ameliorating hypertension [9]. Several successful antihypertensive drugs, such as calcium channel blockers, β-blockers, α-blockers, vasodilators, renin-angiotensin receptor system blockers, and angiotensin-I converting enzyme (ACE) inhibitors, have been used for clinical treatment [10]. ACE, a dipeptidyl cartboxypeptidase, is an important enzyme that converts angiotensin-I to the potent vasoconstrictor, angiotensin-II, which causes blood vessels to narrow, thereby increasing blood pressure [6]. Therefore, the inhibition of ACE is a therapy that significantly decreases hypertension [7].

**Figure 1:**
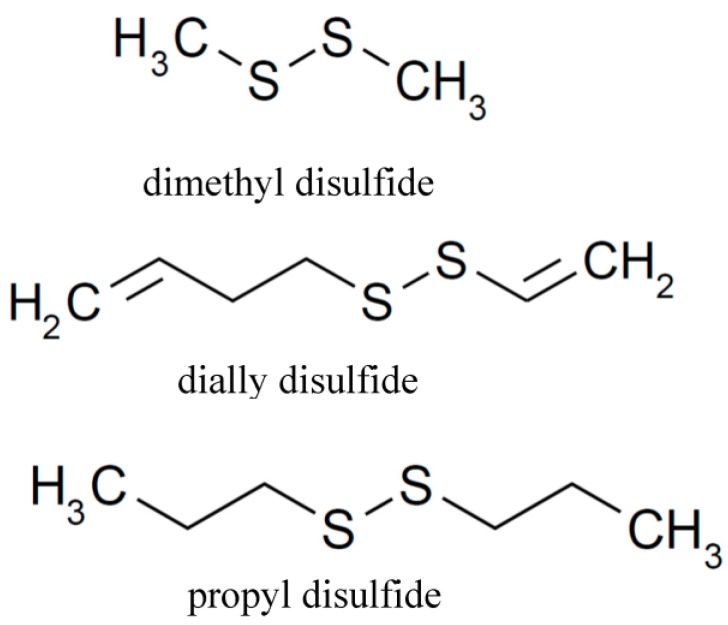
The structure of three organo-sulfur compounds (OSC), DMDS, dimethyl disulfide; DADS, dially disulfide; PDS, propyl disulfide.

There is growing evidence that dietary risks associated with fruits and vegetables are correlated with a lower risk of chronic diseases. Therefore, studies of dietary components, in the absence of anti-inflammatory and antihypertensive drugs, have recently received much attention. Allium species are plants that have been consumed for many centuries. Two widely used Allium species are garlic and onion [10]. Evidence from several studies suggests that the biological and medical benefits of garlic and onion are due to their organo-sulfur compounds (OSC) as ingredients [11]. OSC in garlic or onion contain lipid-soluble components, such as diallyl sulfide (DAS) and diallyl disulfide (DADS), dimethyl disulfide (DMDS), allyl methyl sulfide (AMS), and water-soluble components, such as S-allylcysteine (SAC) and others, which provide, in part, to garlic and onion a unique and characteristic odor and flavor, as well as biological activities [12,13]. The content of DADS, DAS, AMS, and DMDS present in garlic was 530–610, 30–100, 3.8–4.6, and 2.4–2.5 μg/g, respectively [14]. In addition, the content of the major compound in diced onion samples was dipropyl disulfide (PDS), which, with a mean concentration value of 1175.88 μg/g, represented 53% of the total identified compounds in the untreated onion [15]. The biological activity of these individual OSC has been determined. For examples, DAS, DADS, and AMS, have been reported to regulate cytokines and inhibit NO production in stimulated macrophages [16]. Animal studies showed that aged garlic and SAC had anti-hypertensive activity [6]. In addition, DADS may affect adipocyte differentiation through histone acetylation at an early phase of adipocyte differentiation [17]. Aged garlic extract supplementation was effective in lowering the plasma concentration of total cholesterol and low-density cholesterol in hypercholesterolemic men [18]. Moreover, DADS is a histone deacetylase inhibitor, showing cancer chemopreventive properties [19]. We previously reported that DADS, DMDS, and PDS play an important role in the regulation of melanin formation [12]. There is increasing interest in using natural products to produce functional foods because of lower cytotoxicities [16]. Although garlic and onion have been widely investigated for their biological effects, little data on the anti-inflammatory and anti-hypertensive activities of individual bioactive compounds in garlic and onion exists. Therefore, the aim of this study is to explore the effect of the selected dietary organic sulfur compounds, DMDS, DADS, and PDS (Figure 1), on anti-inflammatory activity and vasodilation.

## 2. Results and Discussion

### 2.1. The Effect of the Three OSC on NO Production and the Expression of PGE_2_ in RAW 264.7 Macrophage Cells

**Figure 2:**
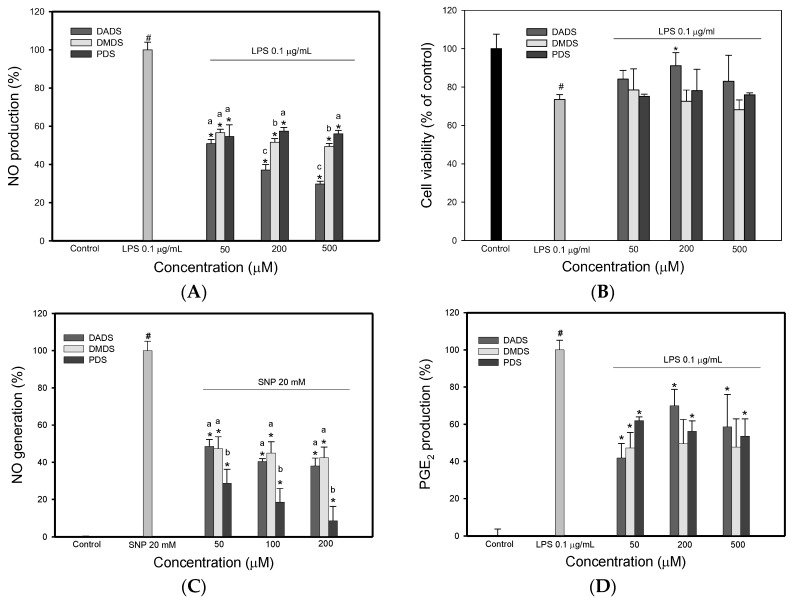
The effects of the three organo-sulfur compounds (OSC) on LPS-induced NO generation, cell survival, scavenging effect on nitric oxide (NO) generation in a cell-free system, and on LPS-induced PGE_2_ production in RAW 264.7 cells. The effect of different organo-sulfur compounds (OSC) on LPS-induced NO production in RAW 264.7 cells. The cells were pretreated with OSC for 6 h, and then LPS (0.1 μg/mL) was added to the medium and incubated for 16 h (**A**); The effect of different organo-sulfur compounds (OSC) on LPS-induced cell survival. The cells were pretreated with OSC for 6 h, and then LPS (0.1 μg/mL) was added to the medium and incubated for 16 h, and then the cell survival was measured by MTT assay (**B**); The scavenging effect of different organo-sulfur compounds (OSC) on nitric oxide (NO) generation by sodium nitroprusside (SNP). SNP and various concentration of OSC were incubated at 25 °C for 1.5 h (**C**); The effect of different organo-sulfur compounds (OSC) on LPS-induced PGE_2_ production in RAW 264.7 cells. The cells were pretreated with OSC for 6 h, and then LPS (0.1 μg/mL) was added to the medium and incubated for 16 h (**D**). DMDS, dimethyl disulfide; DADS, dially disulfide; PDS, propyl disulfide. # *p* < 0.05, compared to the control. * *p* < 0.05, compared to the cells treated with LPS. Values in each concentration with different superscript letters are significantly different (*p* < 0.05).

The ability of the three OSC to influence the NO production in RAW 264.7 macrophages was investigated. The nitrite levels in the culture medium were determined as an index of NO synthesis, using the Griess reagent [20]. As shown in Figure 2A, lipopolysaccharide (LPS)-treated cells produced high levels of nitrite. However, this LPS-induced nitrite production was significantly reduced by incubation with the three OSC at various concentrations. Of the three OSC, DADS was more strongly reduced than DMDS and PDS at 200 and 500 µM. This result is in agreement with previous reports [21] that demonstrated that DADS could attenuate NO production in LPS-induced macrophages. In addition, when the LPS-treated cells were incubated with each OSC, the cell viability was generally >75% at a concentration of 50–500 µM (Figure 2B). The cell viability was also >75% at a concentration of 50–500 µM when cells were treated with the three OSC alone (data not shown). These results imply that the three OSC examined showed no cytotoxicity to RAW 264.7 cells at the tested concentrations. This finding indicates that the reduction of nitrite levels by the three OSC is not due to cell death [22]. Since the three OSC reduced NO production in the LPS-treated cells, it is necessary to determine whether the three OSC scavenge NO directly. Figure 2C shows the scavenging effect of the three OSC on nitric oxide in the sodium nitroprusside (SNP) system. As expected, the three OSC caused a decrease in the production of NO. NO is a short-lived free radical and rapidly reacts with molecular oxygen, to yield nitrogen dioxide, dinitrogen trioxide, and nitrite. Nitrite is the only stable product, so it can be estimated using the Griess reagent. Therefore, scavengers of NO compete with oxygen, leading to a reduction of nitrite generation [23]. According to the data in Figure 2C, the three OSC directly scavenged the NO in a cell-free system, which suggests that the three OSC decrease the NO production in LPS-induced cells because of their propensity to scavenge nitric oxide. Of the three OSC, DADS showed the greatest capacity to scavenge NO.

In order to determine whether the three OSC are capable of inhibiting selected inflammatory mediators, each OSC was assessed for its effect on PGE_2_ production in RAW 264.7 macrophage cells. As shown in Figure 2D, LPS-induced cells produced a high level of PGE_2_, compared to the control (*p* < 0.05). However, the three OSC, in the range of 50–500 µM, demonstrated significant inhibition of PGE_2_ production in LPS-induced macrophage cells. These results show that the three OSC were capable of significantly inhibiting PGE_2_ production at concentrations ranging from 50–500 µM. PGE_2_ is a major lipid mediator of inflammation that is produced from arachidonic acid by the inducible form of cyclooxygenase (COX), COX-2 [24]. The inducible property of PGE_2_ production, when macrophage cells are stimulated by LPS, makes this eicosinoid an ideal target for measuring an inflammatory response in vitro [25]. The results in Figure 2D show that the three OSC significantly inhibited LPS-induced PGE_2_ production, compared to the control, which suggests that the three OSC prevented the LPS-induced inflammatory response. The data in Figure 2A,D show that the three OSC significantly inhibited the production of the LPS-induced inflammatory mediators, PGE_2_ and NO, by RAW 264.7 macrophage cells.

### 2.2. The Effects of the Three OSC on the Expression of iNOS and COX-2 in RAW 264.7 Macrophage Cells

**Figure 3:**
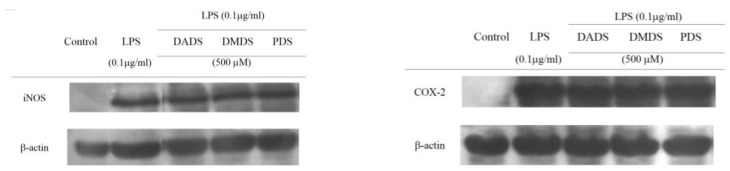

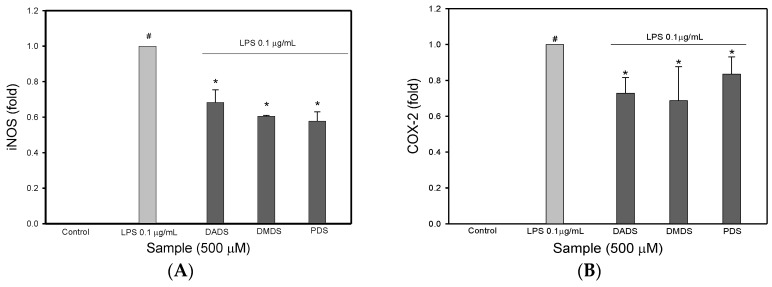
The effect of different organo-sulfur compounds (OSC) on LPS-induced iNOS and COX-2 expression in RAW 264.7 cells. The effect of different organo-sulfur compounds (OSC) on LPS-induced iNOS expression in RAW 264.7 cells. The cells were pretreated with OSC for 6 h, and then LPS (0.1 μg/mL) was added to the medium and incubated for 16 h (**A**); The effect of different organo-sulfur compounds (OSC) on LPS-induced COX-2 expression in RAW 264.7 cells. The cells were pretreated with OSC for 6 h, and then LPS (0.1 μg/mL) was added to the medium and incubated for 16 h (**B**). DMDS, dimethyl disulfide; DADS, dially disulfide; PDS, propyl disulfide. # *p* < 0.05, compared to the control. * *p* < 0.05, compared to the cells treated with LPS. Values in each concentration with different superscript letters are significantly different (*p* < 0.05).

Since the three OSC inhibited NO and PGE_2_ production in stimulated macrophages, this study further examined their effects on iNOS and COX-2 protein in stimulated macrophage cells, in order to clarify the inhibitory mechanism for NO and PGE_2_ production. Figure 3A shows the inhibitory activity of the three OSC on iNOS expression in LPS-induced macrophage cells. The presence of LPS in the culture induced a significant increase in iNOS expression compared with the control. However, when the cells were pretreated with the three OSC, the LPS-induced increase in iNOS expression was significantly suppressed, which indicates that the three OSC may down-regulate the iNOS expression, leading to a reduction in NO production in macrophage cells. Meanwhile, the three OSC at a concentration of 500 µM decreased COX-2 protein expression, although there was no significant difference between DADS, DMDS, and the cells treated with LPS alone (*p* > 0.05) (Figure 3B). Many studies have shown that the production of PGE_2_ and NO by macrophages, upon LPS induction, is mediated by the toll-like receptor 4 (TLR-4) and the subsequent nuclear factor-kB (NF-kB) downstream activation, which results in the expression of COX-2 and iNOS [26]. Therefore, the inhibition of signal transduction for the iNOS and COX-2 gene expression and the direct inhibition of iNOS and COX-2 expression are the main pathways for inhibiting the production of NO and PGE_2_ [27]. Moreover, from the results obtained, it is obvious that the three OSC could attenuate the NO production in LPS-induced macrophage cells, but it is unclear whether the three OSC, as antioxidants, scavenge NO directly. In this study, SNP was used for NO generation in a cell-free system. As expected, the three OSC scavenged the SNP-mediated NO generation (Figure 3C). This finding is meaningful because the three OSC not only showed an inhibitory effect on NO production due to the regulation of iNOS expression, but also scavenged NO directly. This means that the inhibition of NO production by the three OSC might be attributed to a direct NO scavenging effect or an indirect effect resulting from regulating the iNOS expression. In addition, binding of the activated NF-kB to a unique DNA sequence in the iNOS promoter is crucial for the regulation of the iNOS gene expression [21]. Although the influence of the three OSC on the down-regulation of LPS-induced NF-kB activation was not investigated in this study, many reports noted that DADS has been shown to effectively reduce the expression of proinflammatory proteins including iNOS, NF-kB, and matrix metalloproteinase (MMP-9), and it enhances the expression of antioxidant proteins including Nrf-2 (nuclear factor E2-related factor 2) and hemeoxygenase (HO-1) [28]. It was found that LPS-induced iNOS and COX-2 expression in RAW 264.7 macrophage cells was inhibited by the three OSC. This observation explains the inhibition of NO and PGE_2_ production in LPS-induced macrophages, which produces an anti-inflammatory effect. Many iNOS and COX-2 inhibitors are used as anti-inflammatory drugs [5]. The results show that the three OSC, with the inhibition of iNOS and COX-2 expression, demonstrate a good potential for use as anti-inflammatory drugs. However, further in vivo experiments should be explored whether the inhibitory properties of the three OSC on their modulatory properties in inflammatory response contributes to the anti-inflammatory effect of the three OSC.

### 2.3. The Effect of the Three OSC on NO Generation and the Expression of eNOS in SVEC4-10 Cells

**Figure 4:**
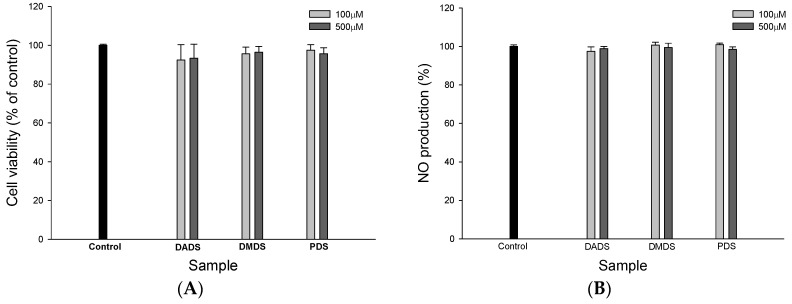

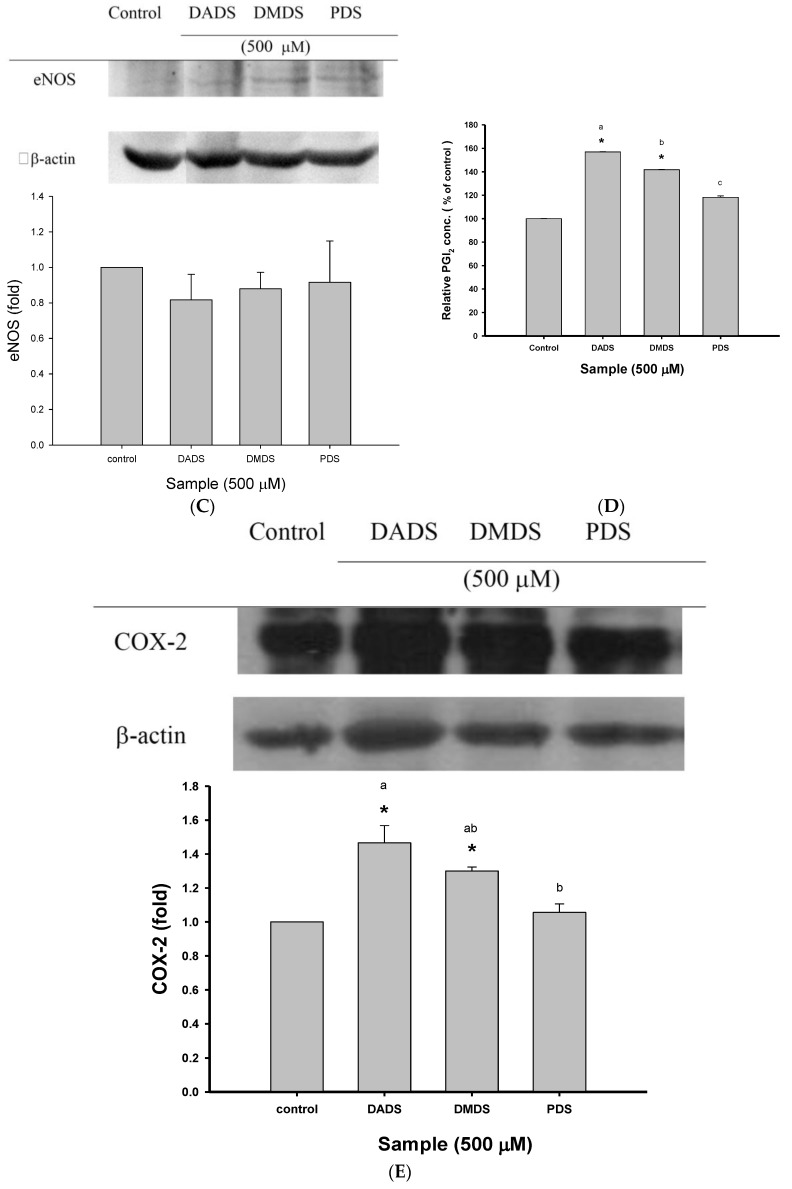
The effects of the three organo-sulfur compounds (OSC) on cell growth, NO production, eNOS expression, PGI_2_ production, and COX-2 expression in SVEC4-10 cells. The effect of different organo-sulfur compounds (OSC) on SVEC4-10 cell growth measured by the MTT assay. The cells were incubated with different organo-sulfur compounds (OSC) for 24 h (**A**); The effect of different organo-sulfur compounds (OSC) on NO production. The cells were incubated with different organo-sulfur compounds (OSC) for 24 h in SVEC4-10 cells (**B**); The effect of different organo-sulfur compounds (OSC) on eNOS expression. The cells were incubated with different organo-sulfur compounds (OSC) for 24 h in SVEC4-10 cells. (**C**); The effect of different organo-sulfur compounds (OSC) on PGI_2_ production. The cells were incubated with different organo-sulfur compounds (OSC) for 4 h in SVEC4-10 cells (**D**); The effect of different organo-sulfur compounds (OSC) on COX-2 expression. The cells were incubated with different organo-sulfur compounds (OSC) for 2 h in SVEC4-10 cells (**E**). DMDS, dimethyl disulfide; DADS, dially disulfide; PDS, propyl disulfide. * *p* < 0.05, compared to the control. Values in each concentration with different superscript letters are significantly different (*p* < 0.05).

In order to understand whether the three OSC have the ability to modulate vasodilation, endothelial cell line SVEC4-10 cells were used. The MTT assay is widely used to determine cell growth. If there was a cytotoxic effect in a test with an OSC, the results for the biological effect of the three OSC would be affected. Therefore, the influence of the three OSC on the viability of SVEC4-10 cells was determined using the MTT assay after 24 h incubation. The results show that at a concentration of 500 µM, none of the three OSC demonstrated any cytotoxic effect on SVEC4-10 cell growth (Figure 4A), which indicates that none of the test concentrations of the three OSC induced cell injury in SVEC4-10 cells.

Nitric oxide is a power vasodilator. Endothelial NOS (eNOS) is a nitric oxide synthase that produces NO in blood vessels. Therefore, we next determined whether the three OSC, at a concentration of 500 µM, modulated NO production and the eNOS expression of SVEC4-10 cells. The results show that the three OSC have no effect on either NO production (Figure 4B) or the eNOS expression (Figure 4C) of SVEC4-10 cells. NO, a freely diffusible gas, has many physiological functions by acting as an intracellular and extracellular messenger. Endothelium-derived NO diffuses into vascular smooth muscle cells (VSMC), eventually causing vasodilation and the alternation of hypertension [6]. In other words, NO deficiency can lead to clinical hypertension [29]. In this study, the three OSC at a concentration of 500 µM did not affect vasodilation by accelerating vascular nitric oxide generation [30].

### 2.4. The Effects of the OSC on PGI_2_ Production and the Expression of COX-2 in SVEC4-10 Cells

Many studies have reported that PGI_2_ plays an important role in attenuating blood pressure [6]. In this study, the effect of the three OSC on PGI_2_ production in SVEC4-10 cells was determined (Figure 4D). Of the three OSC, there was a significant difference (*p* < 0.05) between DADS, DMDS, and the control, which indicated that DADS and DMDS positively induced PGI_2_ production in SVEC4-10 cells. PDS enhanced PGI_2_ production, but no significant difference (*p* > 0.05) was found between PDS and the control. The effect of the three OSC on COX-2 expression is shown in Figure 4E. DADS and DMDS at 500 µM produced a significant increase in COX-2 expression in SVEC4-10 cells, but there was no significant difference between PDS and the control, although PDS enhanced COX-2 expression, indicating that the three OSC positively up-regulated COX-2 expression in SVEC4-10 cells. COX-2 is responsible for the biosynthesis of PGI_2_ from arachidonic acid. Therefore, the results from Figure 4E may explain why both DADS and DMDS induced PGI_2_ generation in SVEC4-10 cells. In addition, COX catalyzes the biosynthesis of various prostaglandins (PGs). After PGI_2_ is liberated from endothelial cells, it binds to the G-protein coupled receptor (Gs) in VSMC, which stimulates adenylate cyclases, which in turn catalyze the production of cyclic adenosine monophosphate (cAMP) from ATP. cAMP increases the activity of cAMP-dependent protein kinase (PKA). Phosphorylation of the K^+^ channel by PKA indirectly leads to a reduced [Ca^2+^]i in VSMC and to a reduction in vascular [6]. In other words, PGI_2_ is believed to be responsible for vasodilative activity. The data in Figure 4E show that DADS, DMDS, and PDS positively up-regulated COX-2 expression, which suggests that DADS, DMDS, and PDS are COX-2 activators in SVEC4-10 cells. In addition, DADS, DMDS, and PDS also increased PGI_2_ production (Figure 4D). These results suggest that the three OSC are potential mediators for vasodilation. 

### 2.5. The Effect of the Three OSC on In Vitro ACE Activity

The effect of the three OSC on in vitro ACE inhibitory activity was evaluated (Table 1). Captopril, a known ACE inhibitor, served as the positive control. Treatment of ACE with the three OSC significantly inhibited ACE activity. The inhibition of ACE was 37.04%, 33.33%, and 25.93% for DADS, DMDS, and PDS, respectively, but the inhibitory effect of the three OSC was significantly lower than that of captopril (1 µM). When the three OSC were added at a concentration of up to 500 µM, the inhibition increased dramatically to 81.48%, 81.48%, and 51.85% for DADS, DMDS, and PDS, respectively. Of the three OSC at a concentration of 500 µM, both DADS and DMDS inhibited ACE activity more than PDS, and there was no significant difference between DADS and DMDS (*p* > 0.05). Both DADS and DMDS at 500 µM inhibited ACE to a greater extent than captopril at 1 µM. These results indicate that DADS, DMDS, and PDS are likely to affect blood pressure via the inhibition of ACE activity.

The renin angiotensin system (RAS) plays a vital role in blood pressure regulation with renin and ACE as the main regulators that control the RAS pathway [31]. ACE is an important enzyme for the control of blood pressure because it catalyzes two different reactions: the conversion of the inactive angiotensin I into a powerful vasoconstrictor and the salt-retainer, angiotensin II, which induces the release of aldosterone that causes the retention of sodium ions by the kidney and elevates blood volume, thus increasing blood pressure [32] and the inactivation of the vasodilator bradykinin, which is conducive to lowering blood pressure [7]. Therefore, by inhibiting ACE activity, the formation of angiotensin II and the destruction of bradykinin are both reduced [33]. As shown in Table 1, the three OSC inhibited ACE activity, in vitro, in a dose-dependent manner, which indicates that the three OSC are useful compounds for the prevention of hypertension. However, this speculation requires further study in vivo.

### 2.6. The Effects of the Three OSC on ROS Generation in SVEC4-10 Cells

**Figure 5:**
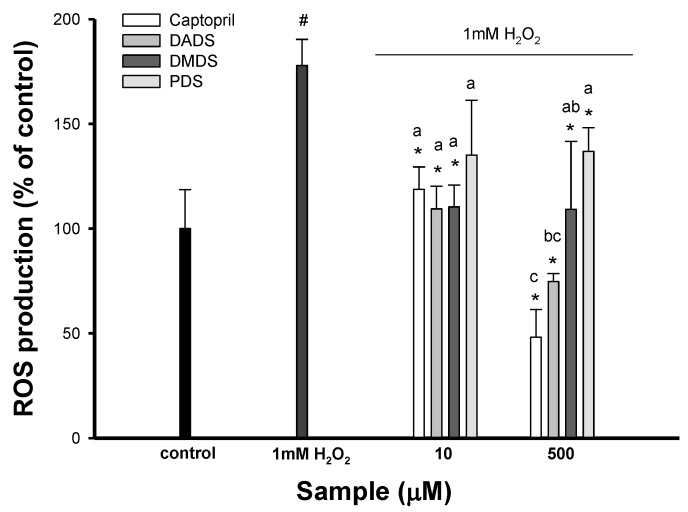
The effect of different organo-sulfur compounds (OSC) on intercellular ROS production in SVEC4-10s induced by 1 mM H_2_O_2_. The data were displayed with mean SD (*n* = 3). # *p* < 0.05 compared with the control and * *p* < 0.05 compared with H_2_O_2_-induced cells alone. Values in each concentration with different superscript letters are significantly different (*p* < 0.05). DMDS, dimethyl disulfide; DADS, dially disulfide; PDS, propyl disulfide.

Many studies have shown that the pathogenesis of hypertension is associated with oxidative stress. In order to understand whether the three OSC display antioxidant activity, the effect of the three OSC on reactive oxygen species (ROS) generation in SVEC4-10 cells induced by 1 mM H_2_O_2_ was investigated. As expected, there was a significant increase in ROS generation of H_2_O_2_-induced SVEC4-10 cells. However, the pretreatment of SVEC4-10 cells with DADS, DMDS, and PDS at 10 and 500 µM decreased ROS generation in H_2_O_2_-induced SVEC4-10 cells (Figure 5). However, PDS at 10 µM had no significant effect on ROS generation in SVEC4-10 cells, compared with H_2_O_2_-induced SVEC4-10 cells. The experimental evidence indicates that ROS play an important pathophysiological role in the development of hypertension. In addition, oxidative stress in cardiac and vascular myocytes is an injury that is caused to cells, resulting from the increased formation of ROS and/or decreased antioxidant reserve [34]. Therefore, antioxidant therapy has beneficial effects on hypertension, due to the scavenging of the free radicals derived from many cell types including endothelial cells, VSMC, and monocytes/macrophages [35]. In this study, DADS, DMDS, and PDS produced a marked reduction in ROS generation, which suggests that their antioxidative properties may in part be responsible for vasodilation. The reduction of ROS formation by antioxidants may attenuate NF-kB activation, which results in decreased eNOS expression, so prolonged antioxidant therapy may attenuate the beneficial regulatory effect of ROS and lead to decreased NO generation [36], which can be seen from the results in Figure 4B that the three OSC had no effect on NO production in SVEC4-10 cells. Accumulating evidence shows that the antihypertensive activity of garlic is probably mediated by one or a combination of biological actions including the inhibition of ACE, a greater reduction in the synthesis of vasoconstrictor prostanoids, eliciting an NO-dependent relaxation, and acting a peroxynitrite radical scavenger [6]. According to the results of the three OSC, DADS and DMDS were significant ACE inhibitors, modulating PGI_2_ formation, up-regulating COX-2 expression, and reducing ROS generation. Therefore, DADS and DMDS may show antihypertensive properties that are probably mediated by their effect on multiple metabolic sites in the regulation of blood pressure [6].

## 3. Materials and Methods

### 3.1. Chemicals

DADS, DMDS, PDS, 2′, 7′-dichlorofluorescin diacetate (DCFH-DA), and ACE from rabbit lung were purchased from Sigma Chemical Co. (St. Louis, MO, USA). Dimethyl sulfoxide (DMSO) was purchased from Aldrich Chemical (Milwaukee, WI, USA). All chemicals were of analytical reagent grade. 

### 3.2. Cell Viability Assay

Cell viability was performed as previously described [37]. The MTT (3-[4, 5-dimethylthiazol-2-yl]-2, 5-dephenyltetrazolium bromide) is a tetrazolium salt and is converted to insoluble formazan by the mitochondrial dehydrogenase of living cells. Briefly, cells were treated and then 5 mg/mL MTT stock solution was added and the cells were incubated for 0.5 h and 2 h for RAW264.7 cells and SVEC4-10 cells, respectively. Subsequently, the reaction was terminated and the plates were incubated for 30 min to solubilize the formazan dye by the addition of DMSO. The optical density of each well was measured with a Thermo Model 355 microplate reader at 550 nm.

### 3.3. Scavenging Activity of OSC on NO

The scavenging effect of OSC on NO was measured according to the method of Marcocci et al. [37]. SNP at physiological pH spontaneously produce NO, which interacts with oxygen to generate nitrite. 20 mM SNP and various concentrations of OSC in PBS (pH 7.4) were incubated at 25 °C for 1.5 h. Then, the nitrite produced from SNP was measured by the Griess reagent (1% sulfanilamide in 5% phosphoric acid and 0.1% *N*-(1-naphthyl)ethylenediamine dihydrochloride in water). 0.1 mL of each supernatant was mixed with the same volume of Griess reagent. The absorbance of the mixture was measured with a Thermo Model 355 microplate reader at 550 nm. 

### 3.4. Inhibitory Action of OSC on NO and PGE_2_ Generation in RAW 264.7 Cells

RAW 264.7 cells (ATCC number: TIB-71) were purchased from the Bioresources Collection and Research Center (Shin-chu, Taiwan) and cultured in Dulbecco’s Modified Eagle’s Medium (DMEM) containing 10% heat-inactivated fetal bovine serum, 1.5 g/L sodium bicarbonate, and 4.5 g/L glucose, and were maintained in humidified 5% CO_2_/95% air at 37 °C. Nitrite levels in the cultured media, which reflect intracellular nitric oxide synthase activity, were determined by the Griess reaction. Briefly, cells were pretreated with OSC for 6 h, and then LPS (0.1 μg/mL) was added to the medium and incubated for 16 h. Then, the growth medium was mixed with the same volume of Griess reagent; absorbance of the mixture at 550 nm was determined by use of an Anthos 2010 microplate reader. For the measurement of PGE_2_ production, the cells were pretreated with OSC for 6 h, and then LPS (0.1 μg/mL) was added to the medium and incubated for 16 h. The PGE_2_ in the cultured media was determined by using the PGE_2_-monoclonal enzyme immunoassay kit (Cayman Chemical, Ann Arbor, MI, USA), according to the manufacturer’s instructions [38]. 

### 3.5. Determination of ACE Activity

The determination of ACE activity was performed using the method of Jimcheena and Gowda [39] with a slight modification. Briefly, the reaction mixture contained 9 μL of potassium phosphate buffer (pH 8.2) which containing 0.3 M NaCl, 15 μL of 5.0 mM hippuryl-histidyl-leucine (HHL), and 30 μL of ACE enzyme solution. The reaction was terminated after incubation at 37 °C for 60 min by the addition of 0.05 mL of 1.0 M HCl. Then, 0.1 mL of pyridine was added, followed by 0.05 mL of benzene sulfonyl chloride (BSC). After mixing for 1 min, the solution was cooled on ice and the absorbance of the mixture was measured with a Thermo Model 355 microplate reader at 405 nm. 

### 3.6. Measurement of NO and 6-keto-PGF1α Generation in SVEC4-10 Cells

Endothelial cell line SVEC4-10 cells (ATCC#CRL-2181, Food Industry Research and Development Institute, Shin-chu, Taiwan) were purchased from Bioresources Collection and Research Center (Shin-chu, Taiwan) and were cultured in DMEM containing 10% heat-inactivated fetal bovine serum, 1.5 g/L sodium bicarbonate, and 4.5 g/L glucose, and were maintained in humidified 5% CO_2_/95% air at 37 °C. Nitrite levels in the cultured media, which reflect intracellular nitric oxide synthase activity, were determined by the Griess reaction. Briefly, cells were cultured with OSC for 24 h. Then, the growth mediums were mixed with the same volume of Griess reagent; absorbance of the mixture at 405 nm was determined by using a Thermo Model 355 microplate reader [38]. As for the measurement of 6-keto-PGF1α production, the cells were cultured with OSC for 4 h, and then 6-keto-PGF1α (stable metabolite of prostacyclin, PGI_2_) levels were measured using 6-keto-PGF1α Immunoassay kits (Cayman Chemical, Ann Arbor, MI, USA), according to the manufacturer’s instructions. 

### 3.7. Western Blot

The RAW264.7 macrophage cells were treated with OSC for 6 h, and then LPS (0.1 μg/mL) was added to the medium and incubated for 16 h. As for SVEC4-10 cells, the cells were cultured with OSC for 24 h and 2 h for eNOS and COX-2 expression, respectively. The cells were washed with ice-cold phosphate buffer saline (PBS), and then the cells were treated with lysis buffer. Cellular lysates were centrifuged at 10,000× *g* at 4 °C for 20 min. The supernatants were collected and the protein contents were determined by using the BCA protein assay kit (Piece, Pockfold, IL, USA). Each sample, which contained 50 μg protein, was separated on 8% sodium dodecyl sulfate (SDS)–polyacrylamide gels. After electrophoresis, gels were transferred to a nitrocellulose membrane [40]. After washing with distilled water, the membrane was incubated with 5% albumin in PBST (0.1% Tween-20 in PBS, pH 7.4) for 30 min and was then immunoblotted with mouse monoclonal anti-iNOS antibody (Santa Cruz, CA, USA), or rabbit polyclonal anti-COX-2 antibody (Cell Signaling, Danvers, MA, USA), or anti-eNOS antibody (Cell Signaling, Danvers, MA, USA), or mouse monoclonal anti-Tubulin antibody (Santa Cruz, CA, USA). Blots were then incubated with anti-mouse or anti-rabbit immunoglobulin G (IgG) antibody conjugated to horseradish peroxidase (Santa Cruz, CA, USA). Binding was detected by chemiluminescence, captured on Kodak XAR-5 film (Eastman Kodak, Rochester, NY, USA), and visualized using an enhanced chemiluminescence (ECL) kit (Amersham, Piscataway, NJ, USA) [38]. 

### 3.8. Evaluation of ROS

To determine the generation of ROS in cells, 2′,7′-dichlorofluorescein-diacetate (DCFH-DA) was used, when it penetrates the cell membranes and is hydrolyzed by intracellular esterase to form dichlorodihydro-fluorescein (DCFH) [41]. DCFH reacted with ROS generated by intracellular stress to produce highly fluorescent DCF which emitted fluorescence when excited at 485 nm. The SVEC4-10 cells were treated with different OSC for 15 min and were then exposed to 1 mM H_2_O_2_ for 15 min. Subsequently, DCFH-DA (50 μM) was added to the medium and incubated for 90 min. The treatment medium was removed, and the cells were washed twice with PBS. The ROS produced from intracellular stress was detected using a Bio-Tek FLx800 microplate fluorescence reader (Winoosky, VT, USA) with an excitation wavelength of 485 nm and an emission wavelength of 530 nm.

### 3.9. Statistical Analysis

All data were presented as means ± SD. Statistical analysis involved the use of the Statistical Analysis System software package. Analysis of variance was performed by ANOVA procedures. Significant differences between means were determined by Duncan’s multiple range tests at a level of *p* < 0.05.

## 4. Discussion

In summary, these results indicate that the three OSC exhibited significant anti-inflammatory activity by LPS-induced macrophages, which suggests that DMDS, DADS, and PDS played an important role in regulating the cell-mediated immune response. In addition, the up-regulation of the vasodilating pathways and the antioxidant potential by DMDS, DADS, and PDS provides new insights into the properties of the three OSC. Overall, the results suggest that the three OSC are potential anti-inflammatory and vasodilative mediators. However, scientific trials in vivo are needed to confirm the results.

## Figures and Tables

**Table 1:** The effect of dimethyl disulfide (DMDS), dially disulfide (DADS), and propyl disulfide (PDS) on angiotensin converting enzyme (ACE) activity.

Sample	Inhibition (%)	
	100 µM	500 µM
**DADS**	37.04 ± 0.26 ^b^	81.48 ± 0.16 ^a^
**DMDS**	33.33 ± 0.11 ^b^	81.48 ± 0.05 ^a^
**PDS**	25.93 ± 0.31 ^b^	51.85 ± 0.26 ^b^
**Captopril (1 µM)**	74.07 ± 0.38 ^a^	

Results are expressed as the percentage of the control, and the data were displayed with mean ± SD (*n* = 3). Mean values with different letters in each column are statistically significant (*p* < 0.05).
